# DEAD-Box Protein Ddx46 Is Required for the Development of the Digestive Organs and Brain in Zebrafish

**DOI:** 10.1371/journal.pone.0033675

**Published:** 2012-03-19

**Authors:** Shunya Hozumi, Ryo Hirabayashi, Akio Yoshizawa, Mitsuko Ogata, Tohru Ishitani, Makiko Tsutsumi, Atsushi Kuroiwa, Motoyuki Itoh, Yutaka Kikuchi

**Affiliations:** 1 Department of Biological Science, Graduate School of Science, Hiroshima University, Higashi-Hiroshima, Hiroshima, Japan; 2 Division of Biological Science, Graduate School of Science, Nagoya University, Chikusa-ku, Nagoya, Japan; Temasek Life Sciences Laboratory, Singapore

## Abstract

Spatially and temporally controlled gene expression, including transcription, several mRNA processing steps, and the export of mature mRNA to the cytoplasm, is essential for developmental processes. It is well known that RNA helicases of the DExD/H-box protein family are involved in these gene expression processes, including transcription, pre-mRNA splicing, and rRNA biogenesis. Although one DExD/H-box protein, Prp5, a homologue of vertebrate Ddx46, has been shown to play important roles in pre-mRNA splicing in yeast, the *in vivo* function of Ddx46 remains to be fully elucidated in metazoans. In this study, we isolated zebrafish *morendo* (*mor*), a mutant that shows developmental defects in the digestive organs and brain, and found that it encodes Ddx46. The *Ddx46* transcript is maternally supplied, and as development proceeds in zebrafish larvae, its ubiquitous expression gradually becomes restricted to those organs. The results of whole-mount *in situ* hybridization showed that the expression of various molecular markers in these organs is considerably reduced in the *Ddx46* mutant. Furthermore, splicing status analysis with RT-PCR revealed unspliced forms of mRNAs in the digestive organ and brain tissues of the *Ddx46* mutant, suggesting that Ddx46 may be required for pre-mRNA splicing during zebrafish development. Therefore, our results suggest a model in which zebrafish Ddx46 is required for the development of the digestive organs and brain, possibly through the control of pre-mRNA splicing.

## Introduction

Precursor mRNA (pre-mRNA) splicing is essential for gene expression in metazoan cells, and the splicing reaction proceeds via a coordinated series of RNA-RNA, RNA-protein, and protein-protein interactions, which lead to exon ligation and the release of the intron lariat [1]–[Bibr pone.0033675-Brow1] [Bibr pone.0033675-Smith1] [4]. Pre-mRNA splicing is catalyzed by the macromolecular machinery known as the spliceosome, which consists of five small nuclear ribonucleoprotein particles (snRNPs: U1, U2, U4, U5, and U6) and >150 proteins. Non-snRNP proteins, which belong to a group of DExD/H-box RNA-dependent ATPases/helicases, are required for the pre-mRNA splicing process in yeast [1]–[Bibr pone.0033675-Brow1] [Bibr pone.0033675-Smith1] [4].

The DExD/H-box RNA helicase family is a large protein group characterized by the presence of a helicase domain that is highly conserved from bacteria to humans [5]–[Bibr pone.0033675-Rocak1] [Bibr pone.0033675-Bleichert1] [8]. The DExD/H-box helicases share nine conserved motifs; motifs Q, I, II, and VI are required for NTP/ATP binding and catalyze its hydrolysis [5]–[Bibr pone.0033675-Rocak1] [Bibr pone.0033675-Bleichert1] [8]. These proteins have been shown to play important roles in all aspects of RNA metabolism, including the modulation of RNA structures and association/dissociation of RNA-protein complexes, such as pre-mRNA splicing, rRNA biogenesis, transcription, RNA stability and turnover, RNA export, and translation [5]–[Bibr pone.0033675-Rocak1] [Bibr pone.0033675-Bleichert1] [8]. In the yeast *Saccharomyces cerevisiae*, eight DExD/H-box proteins-Sub2, Prp5, Prp28, Brr2, Prp2, Prp16, Prp22, and Prp43-act in specific steps of the splicing cycles to catalyze RNA-RNA rearrangements and RNP remodeling [2]–[Bibr pone.0033675-Smith1] [4]. Among them, Prp5 (a homologue of vertebrate Ddx46) is necessary, along with ATP hydrolysis, for stable association of U2 snRNP with pre-mRNA and pre-spliceosome formation in *S. cerevisiae* and *Schizosaccharomyces pombe* [9]–[Bibr pone.0033675-Xu1] [11]. In addition, human DDX46 has been shown to play roles in pre-mRNA splicing *in vitro* before or during prespliceosome assembly [12]. The *in vivo* function of Ddx46 in metazoans remains to be elucidated, however.

The zebrafish has emerged as an important model system for the investigation of vertebrate development and other complex biological processes, including human disease [13], [14]. Analyses of zebrafish mutants and knock-down embryos have provided significant insights into the *in vivo* function of the genes responsible for the mutants or the targeting genes [13], [14]. Here, we discuss the function of Ddx46 in the development of the digestive organs and brain using a newly identified zebrafish *Ddx46* mutant, *morendo* (*mor*). *Ddx46* is expressed maternally and ubiquitously, and its expression gradually becomes restricted to the digestive organs and brain. Phenotypic analysis of the *Ddx46* mutant and the examination of various molecular marker expressions using whole-mount *in situ* hybridization of the digestive organs and brain showed that zebrafish Ddx46 is required for the development of these organs. Based on RT-PCR analyses, we propose that Ddx46 plays a role in pre-mRNA splicing in the digestive organs and brain during zebrafish development.

## Results

### The *mor^ha4^* mutant has defects in the development of the digestive organs and brain

**Figure 1:**
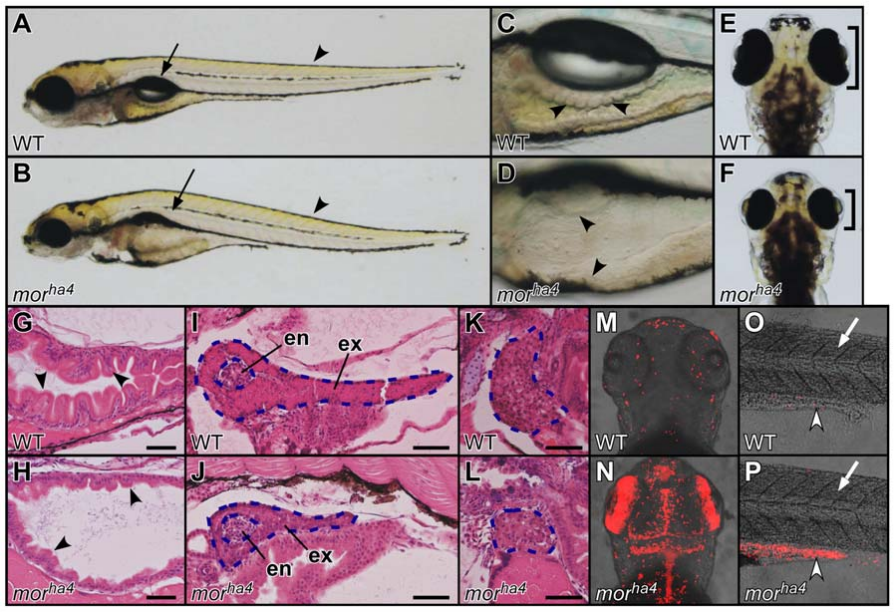
Phenotype of the *mor^ha4^* mutant. (A–F) Lateral (A–D) and dorsal (E, F) views of live WT and *mor^ha4^* larvae at 5.5 dpf. The swim bladder failed to inflate (arrows in A, B), the intestine lacked folds (arrowheads in C, D), and the retinae were reduced in size (brackets in E, F) in the *mor^ha4^* mutant. Conversely, somite formation in the *mor^ha4^* mutant appeared normal (arrowheads in A, B). (G–L) Sagittal sections of 5.5-dpf larvae stained with hematoxylin and eosin. The intestine lacked folds and was thin walled (arrowheads in G, H), and the exocrine pancreas (blue dotted lines in I, J) and liver (blue dotted lines in K, L) were small in the *mor^ha4^* mutant. In contrast, the endocrine pancreas (blue dotted lines in I, J) in WT larvae was indistinguishable from that in *mor^ha4^* larvae. Scale bars, 50 µm. (M–P) Dorsal views, anterior to the top (M, N). Lateral views, anterior to the left (O, P). Apoptotic cells were detected using the TUNEL method. An increase in apoptotic cells was evident in the brain, retinae, and posterior intestine of the *mor^ha4^* larvae (white arrowheads in O, P) compared to WT larvae, but not in the *mor^ha4^* somite (white arrows in O, P). en, endocrine pancreas; ex, exocrine pancreas.

To elucidate the mechanisms that underlie the formation of the intestinal epithelium during development, we took a forward genetic approach. One mutant that we identified, *mor^ha4^*, had defects in intestinal epithelium and retinal development, and showed a recessive larval lethal phenotype. Phenotypic analyses of the *mor^ha4^* mutant revealed that the swim bladder failed to inflate (Figure 1A–D), the intestine lacked folds (Figure 1C, D, G, and H), and the retinae were smaller than normal (Figure 1E and F) at 5.5 days post fertilization (dpf). In addition, histochemical and immunohistochemical analyses exhibited that the exocrine pancreas and liver in the *mor^ha4^* mutant were smaller than those in wild-type (WT) larvae (Figure 1I–L, Figure S1), whereas the size of the endocrine pancreas was normal in this mutant (Figure 1I and J). We also found that cell death was increased in the brain, retinae, and intestine in the *mor^ha4^* mutant but not in the WT at 3 dpf (brain and retinae) or 5 dpf (intestine) (Figure 1M–P). Conversely, the formation of somite was apparently unaffected (Figure 1A and B), and increased cell death was not detected in the *mor^ha4^* somite at 5 dpf (Figure 1O and P). These results suggest that the *mor^ha4^* mutant has defects in digestive organ and brain development.

### The *mor* locus encodes Ddx46

**Figure 2:**
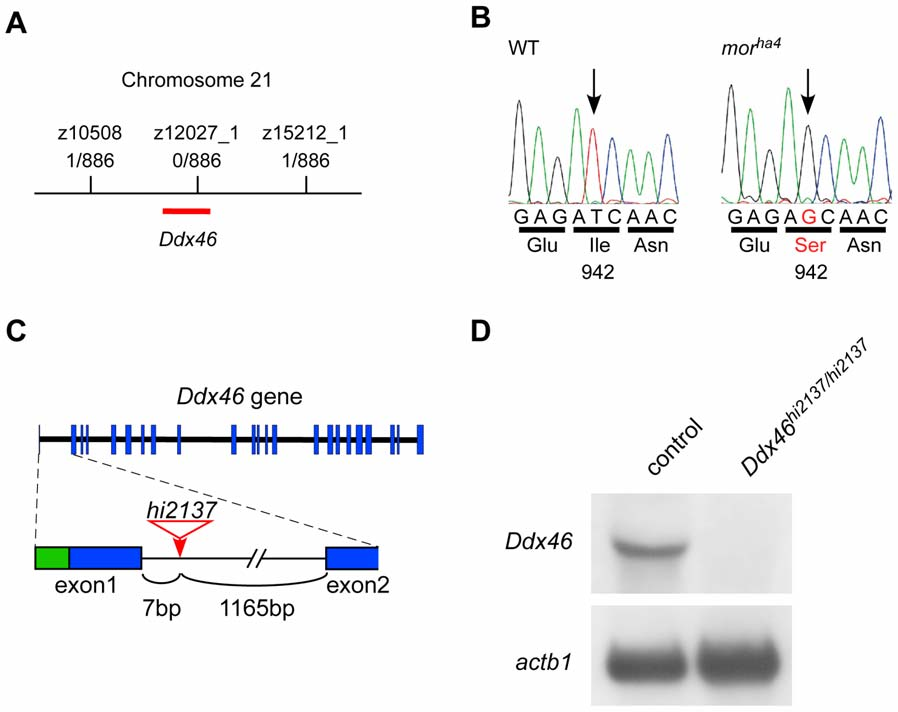
Identification of the *mor* gene and analysis of the *hi2137* allele. (A) Meiotic and physical map schematic of the *mor* locus on chromosome 21. The number of recombinants and larvae genotyped is shown for each microsatellite marker. (B) Sequencing cDNA from WT and *mor^ha4^* larvae revealed a nucleotide exchange from T to G, which resulted in an Ile-to-Ser transition at amino acid 942 in the *mor^ha4^* mutant. (C) Genomic structure of the *Ddx46* gene showing the viral insertion site in the *hi2137* allele (red). Exons are boxes, with coding and non-coding sequences in blue and green, respectively. The viral insertion (red arrow) occurs in the first intron between exons 1 and 2. (D) Northern blot analysis of *Ddx46^hi2137/hi2137^* mutants and control larvae at 3.5 dpf. No *Ddx46* transcript was found in the *Ddx46^hi2137/hi2137^* mutants, whereas the level of *actb1* transcript in the mutants was the same as that in control larvae. Control larvae were sibling WT or *Ddx46^hi2137/+^* larvae and had normal phenotypes.

The *mor^ha4^* mutation was meiotically mapped to a region of chromosome 21 defined by two microsatellites, z10508 and z15212_1, in the Zv6 ensemble assembly of the zebrafish genome (Figure 2A). At this point, we learned that the *Ddx46* mutation (*Ddx46^hi2137^*), which was isolated using a large insertional screening [15] and causes a similar phenotype in *mor^ha4^* (http://web.mit.edu/hopkins/group11.html), was also positioned on the same region of chromosome 21 (see Figure 2A). Given the similarities between *Ddx46^hi2137^* and *mor^ha4^*, we attempted to position the *Ddx46* gene in relation to the *mor* locus. No recombination was observed between the *mor^ha4^* phenotype and a *Ddx46* intronic polymorphic marker, z12027_1 (see Figure 2A). Thus, both mapping and the phenotype of the *Ddx46^hi2137^* mutant suggested that *Ddx46* is a good candidate for the *mor^ha4^* mutation. To see whether *mor^ha4^* is a mutation of the *Ddx46* gene, *Ddx46* cDNA was cloned and sequenced from WT and mutant embryos. Sequencing of the *mor^ha4^* mutant revealed a T-to-G transversion, which introduced a serine in place of an isoleucine at amino acid position 942 in the C-terminal region of the Ddx46 protein (Figures 2B and S2). The sequence alignment of the human, mouse, chicken, and zebrafish Ddx46 proteins showed a high level of conservation in the C-terminal region among these vertebrates (see Figure S2). We confirmed that this lesion segregated with the mutant phenotypes in 200 meiotic events (data not shown).

Although the viral insertion site of the *Ddx46^hi2137^* mutant was identified in intron 1 of the *Ddx46* gene (Figure 2C; http://web.mit.edu/hopkins/group11.html), no *Ddx46* transcript was detected in the *Ddx46^hi2137/hi2137^* mutants at 3.5 dpf (Figure 2D). These data indicated that the viral insertion strongly abrogates the transcription of *Ddx46* or transcript stability, as observed previously [16]. To confirm that the loss of Ddx46 function accounted for the *mor^ha4^* phenotype, we performed complementation analysis between the *mor^ha4^* and *Ddx46^hi2137^* alleles. In transheterozygote (*mor^ha4^*/*Ddx46^hi2137^*) larvae, the swim bladder failed to inflate, the intestine lacked folds, and the retinae were smaller than normal-the same phenotype of the *mor^ha4^* mutant (see Figure S3).

**Figure 3:**
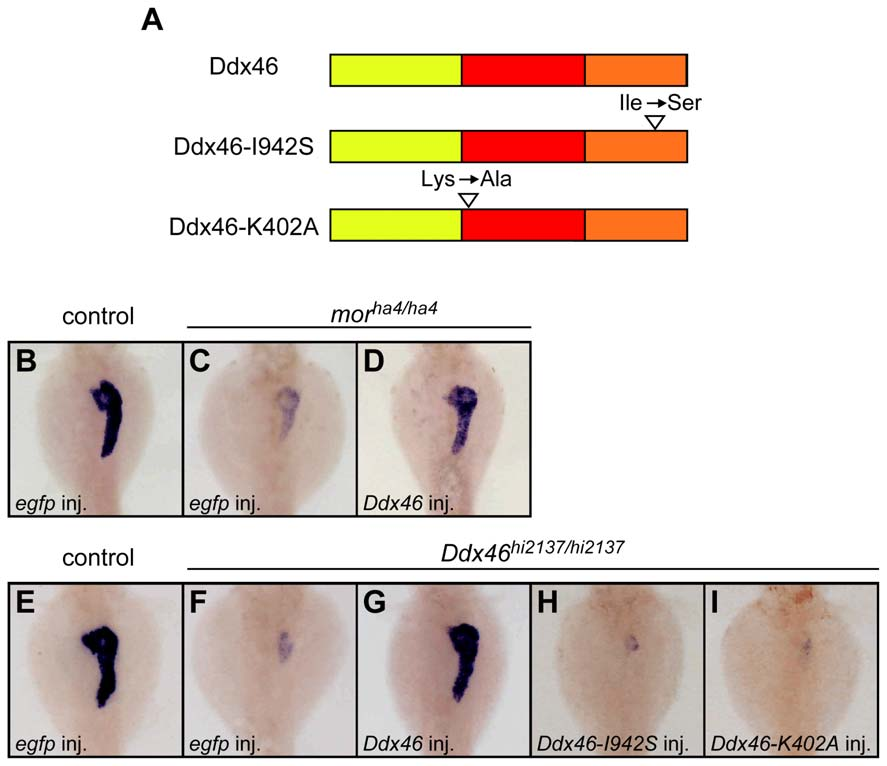
Defects of exocrine pancreas formation in both *mor^ha4/ha4^* and *Ddx46^hi2137/hi2137^* mutants are rescued by the overexpression of *Ddx46* mRNA but not mutated *Ddx46* mRNA. (A) Scheme of the Ddx46 protein structure. The yellow, red, and orange boxes indicate the N-terminal, DEAD-box helicase, and C-terminal domain, respectively. Mutations were introduced into the Ddx46 protein; in Ddx46-I942S, an isoleucine in the C-terminal domain of Ddx46 was changed to serine, which is the same mutation as that in the *mor^ha4^* mutant; in Ddx46-K402A, GKT in motif I, which is important for ATPase activity in Ddx46 homologues, was changed to GAT. (B–I) All dorsal views, anterior to the top. The expression of *try*, a molecular marker for the exocrine pancreas, was examined using whole-mount *in situ* hybridization at 3.5 dpf. The *try* expression in the exocrine pancreas was markedly reduced in *egfp* mRNA-injected *mor^ha4/ha4^* (C) and *Ddx46^hi2137/hi2137^* mutants (F) compared to *egfp* mRNA-injected control larvae (B, E). The *try* expression was rescued in the *Ddx46* mRNA-injected *mor^ha4/ha4^* (D) and *Ddx46^hi2137/hi2137^* mutants (G), whereas no rescue was achieved by the overexpression of *Ddx46-I942S* (H) or *Ddx46-K402A* (I) mRNA into *Ddx46^hi2137/hi2137^* mutants. Control larvae-sibling WT or *mor^ha4/+^* larvae (B–D), sibling WT or *Ddx46^hi2137/+^* larvae (E–I)-had normal phenotypes.

We next performed rescue experiments using both alleles (*mor^ha4^* and *Ddx46^hi2137^*). As observed with histological section data, the size of the exocrine pancreas, which is detected through *trypsin* (*try*) [17] expression, was markedly reduced in *egfp* mRNA-injected *mor^ha4/ha4^* mutants (17 of 18 *egfp* mRNA-injected *mor^ha4/ha4^* mutants reduced) compared to *egfp* mRNA-injected control larvae (0 of 22 *egfp* mRNA-injected control larvae reduced; Figure 3B and C). We found that the expression of *try* in the *mor^ha4/ha4^* mutant was rescued by the overexpression of *Ddx46* mRNA (12 of 12 *Ddx46* mRNA-injected *mor^ha4/ha4^* mutants rescued; Figure 3B–D). As in *mor^ha4/ha4^* mutants, the size of the exocrine pancreas was also markedly reduced in *egfp* mRNA-injected *Ddx46^hi2137/hi2137^* mutants (16 of 16 *egfp* mRNA-injected *Ddx46^hi2137/hi2137^* mutants reduced) compared to *egfp* mRNA-injected control larvae (0 of 20 *egfp* mRNA-injected control larvae reduced; Figure 3E and F). We found that the overexpression of *Ddx46* mRNA in the *Ddx46^hi2137/hi2137^* larvae rescued the size of the exocrine pancreas (11 of 11 *Ddx46* mRNA-injected *Ddx46^hi2137/hi2137^* mutants rescued; Figure 3E and G). Our results showed that the defects of the pancreas in both *mor^ha4/ha4^* and *Ddx46^hi2137/hi2137^* larvae were rescued by the overexpression of *Ddx46* mRNA. Together, genetic data, complimentation analysis, and rescue experiments indicated that the *mor* gene corresponds to *Ddx46*.

### Effect of the *mor^ha4^* point mutation on Ddx46 function

To investigate the effect of the *mor^ha4^* point mutation on Ddx46 function, we also performed rescue experiments using the *mor^ha4^* mutant gene *Ddx46-I942S* (Figure 3A). The expression of *try* in the *Ddx46^hi2137/hi2137^* mutant was not rescued by the overexpression of *Ddx46-I942S* mRNA (0 of 21 *Ddx46-I942S* mRNA-injected *Ddx46^hi2137/hi2137^* mutants rescued; Figure 3H). This result suggested that the function of Ddx46 is abolished by the *mor^ha4^* point mutation. Moreover, to investigate the importance of the ATPase activity of Ddx46 to its function in zebrafish larvae, we introduced a mutation into motif I of the DEAD box (see Figure 3A; substitution from lysine to alanine at amino acid position 402), which is known to disrupt ATPase activity in *S. pombe* Prp5 [10]. Overexpression of *Ddx46-K402A* mRNA in the mutant larvae failed to rescue the size of the exocrine pancreas (0 of 26 *Ddx46-K402A* mRNA-injected *Ddx46^hi2137/hi2137^* mutants rescued; Figure 3I), suggesting that the ATPase activity of Ddx46 is necessary for it to function in zebrafish development.

### *Ddx46* expression is restricted to developing digestive organs and brain

**Figure 4:**
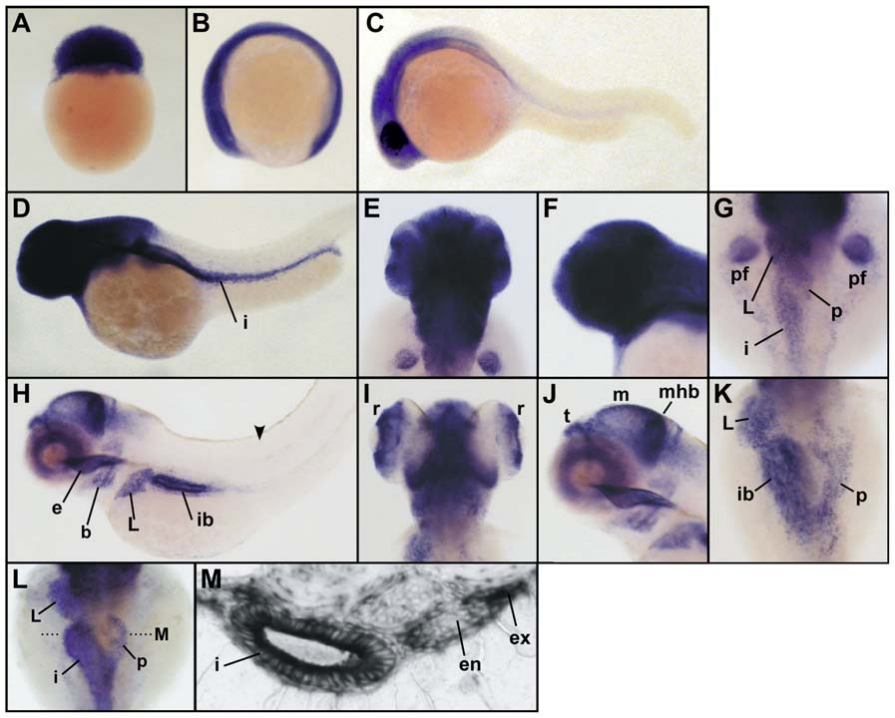
*Ddx46* expression in the developing zebrafish. (A–K) *Ddx46* expression was examined using whole-mount *in situ* hybridization in WT embryos or larvae at the 128-cell (A), 6-somite (B), 1-dpf (C), 2-dpf (D–G), and 4-dpf (H–K) stages. Lateral view, animal pole to the top (A). Lateral views, anterior to the left (B, C, D, F, H, J). Dorsal views, anterior to the top (E, G, I, K). The *Ddx46* transcript was maternally supplied and continued to be expressed ubiquitously during the somitogenesis stages (A, B). By 1 dpf, *Ddx46* expression became restricted to the head region (C). At 2 dpf, strong *Ddx46* expression was prominent in the head, pectoral fin bud, and digestive organs (D–G). At 4 dpf, *Ddx46* expression was further restricted to the retina, telencephalon, midbrain, midbrain-hindbrain boundary, branchial arch, esophagus, liver, pancreas, and intestinal bulb (H–K). No *Ddx46* transcript was detected in the somite (arrowhead in H). (L, M) *Ddx46* expression was examined using whole-mount *in situ* hybridization in WT larvae at 3 dpf. Dorsal views, anterior to the top (L). A transverse section was cut at the level indicated by the black dotted line in L. The section revealed *Ddx46* expression in the intestine and exocrine pancreas, but not in the endocrine pancreas (M). b, branchial arches; e, esophagus; en, endocrine pancreas; ex, exocrine pancreas; i, intestine; ib, intestinal bulb; L, liver; m, midbrain; mhb, midbrain-hindbrain boundary; p, pancreas; pf, pectoral fin bud; r, retina; t, telencephalon.

To define the spatiotemporal expression of *Ddx46* in developing embryos and larvae, we performed whole-mount *in situ* hybridization. *Ddx46* was found to be a maternally supplied transcript that was expressed ubiquitously during early somitogenesis (Figure 4A and B). Its expression became restricted to the head region by 24 hours post-fertilization (hpf) (Figure 4C). By 2 dpf, *Ddx46* was expressed in the head, retina, digestive organs, and pectoral fin bud (Figure 4D–G), and at 4 dpf, its expression was even more confined to the retinae, telencephalon, midbrain, midbrain-hindbrain boundary, branchial arches, esophagus, liver, pancreas, and intestinal bulb (Figure 4H–K). Transverse section data revealed the presence of the *Ddx46* transcript in pancreatic exocrine cells but not in pancreatic endocrine cells (Figure 4L and M). Further, we found that *Ddx46* transcripts were not present in the somite after 4 dpf (see Figures 4H). These *Ddx46* expression patterns were consistent with nearly all aspects of the *mor^ha4^* mutant phenotype.

### Gene expression in the digestive organs and brain is down-regulated in the *Ddx46^hi2137/hi2137^* mutants

**Figure 5:**
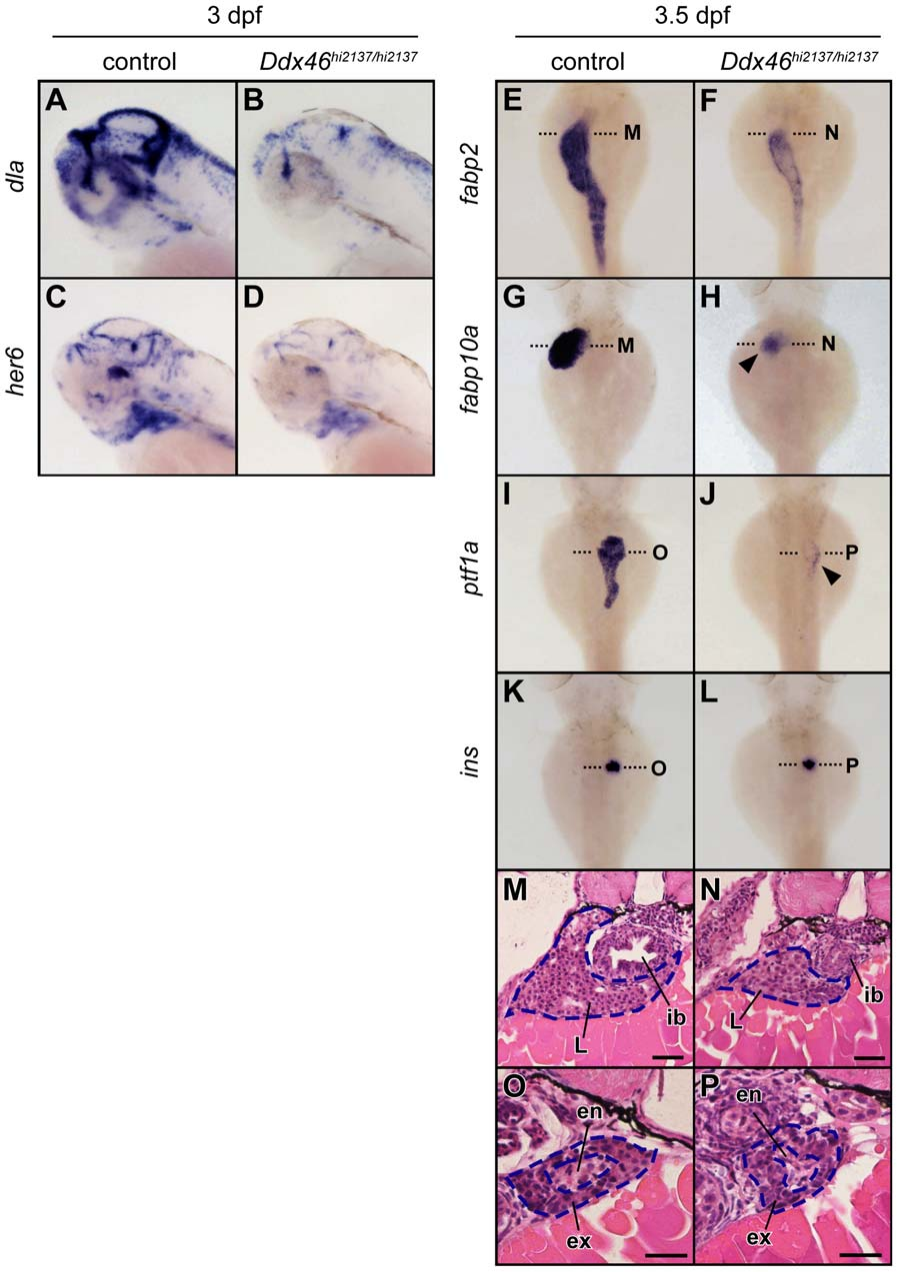
Expression of molecular markers for digestive organs and brain is reduced in the *Ddx46^hi2137/hi2137^* mutant. (A–D) The expression of *dla* and *her6* was examined using whole-mount *in situ* hybridization at 3 dpf. All lateral views, anterior to the left. (E–L) The expression of *fabp2*, *fabp10a*, *ptf1a*, and *ins* was examined using whole-mount *in situ* hybridization at 3.5 dpf. All dorsal views, anterior to the top. In the *Ddx46^hi2137/hi2137^* mutants, the intensity and area of *dla*, *her6*, *fabp2*, *fabp10a*, and *ptf1a* expression were markedly reduced at 3 or 3.5 dpf (A–J; arrowheads in H, J). In contrast, the *ins* expression in the *Ddx46^hi2137/hi2137^* mutant did not change at these developmental stages (K, L). (M–P) Transverse sections of 3.5-dpf *Ddx46^hi2137/hi2137^* mutant larvae stained with hematoxylin and eosin. The transverse sections were cut at the levels indicated by black dotted lines in E–L. The tissues in the intestinal bulb, liver, and exocrine pancreas were still present in the *Ddx46^hi2137/hi2137^* mutant larvae at 3.5 dpf. Scale bars, 50 µm. en, endocrine pancreas; ex, exocrine pancreas; ib, intestinal bulb; L, liver. Control larvae were sibling WT or *Ddx46^hi2137/+^* larvae and had normal phenotypes.

We showed that the *Ddx46* mutant displays defects in the development of the digestive organs and brain. To explore these defects during development, we examined the expression of various molecular markers using whole-mount *in situ* hybridization. At 2.5 dpf, the expression level and pattern of *foxa3* [18] of the control and *Ddx46^hi2137/hi2137^* larvae were indistinguishable (Figure S4), indicating that the budding of the digestive organs was normal in the *Ddx46^hi2137/hi2137^* mutants. The expressions of *deltaA* (*dla*) [19], [20], and *her6* [21] in the brain or retinae were markedly reduced in the *Ddx46^hi2137/hi2137^* larvae at 3 dpf, however (Figure 5A–D). In addition, we found that the expressions of intestinal epithelium marker *fabp2* [22], liver marker *fabp10a* [23], and exocrine pancratic marker *pancreas specific transcription factor, 1a* (*ptf1a*) [24] were also markedly reduced in the *Ddx46* mutants at 3.5 dpf (Figures 5E–5J). In contrast, expressions of endocrine pancreatic marker *preproinsulin* (*ins*) [25] and a myogenesis marker of the somite, *myogenic differentiation 1* (*myod1*) [26], did not change in the *Ddx46^hi2137/hi2137^* mutant (Figure 5K and L; Figure S5). Consistent with this result, *Ddx46* was not expressed in pancreatic endocrine tissues (Figure 4M) or the somite (Figure 4H). We also examined the expression of various molecular markers in the *mor^ha4/ha4^* mutant. Downregulation of the expression levels of *dla*, *fabp2*, *fabp10a*, and *ptf1a* in the *mor^ha4/ha4^* mutant was less severe than that in the *Ddx46^hi2137/hi2137^* mutant (Figure 5, Figure S6), suggesting that *mor^ha4^* is a hypomorphic allele.

We next tested whether the down-regulation of these mRNAs is due to the loss of tissues in the liver and exocrine pancreas in the *Ddx46^hi2137/hi2137^* mutant. Transverse section data of the *Ddx46^hi2137/hi2137^* mutant showed that although the size of the liver and exocrine pancreas is smaller than normal, the tissues of these organs are still present at 3.5 dpf (Figure 5M–P). These results suggested that the amount of mRNAs in these organs is reduced specifically in this mutant.

We also examined the expressions of molecular markers such as *dla*, *fabp10a*, *ptf1a*, and *ins* in transheterozygote (*mor^ha4^*/*Ddx46^hi2137^*) larvae at 3 or 3.5 dpf, and found that, with the exception of *ins*, they were markedly reduced (Figure S7), as observed in the *Ddx46^hi2137/hi2137^* mutants. These results further supported the conclusion that the *mor* gene corresponds to *Ddx46*.

Furthermore, we found that the expressions of other molecular markers for the digestive organs and brain- *her4* (brain and retina) [27], [28], *neurogenin 1* (*neurog1*: brain) [29], *neurod* (brain and retina) [29], *homeo box HB9 like a* (*hlxb9la*: exocrine pancreas) [30], *carboxypeptidase A5* (*cpa5*: exocrine pancreas) [31], *gata6* (intestine, liver, and exocrine pancreas) [32], and *dehydrogenase/reductase member 9* (*dhrs9*: intestine and liver) [33]-were markedly reduced in the *Ddx46^hi2137/hi2137^* larvae from 3 to 3.5 dpf (Figure S8). These results suggested that Ddx46 is required for gene expression in the digestive organs and brain.

### *Ddx46^hi2137/hi2137^* mutant has defects in pre-mRNA splicing in the digestive organs and brain

**Figure 6:**
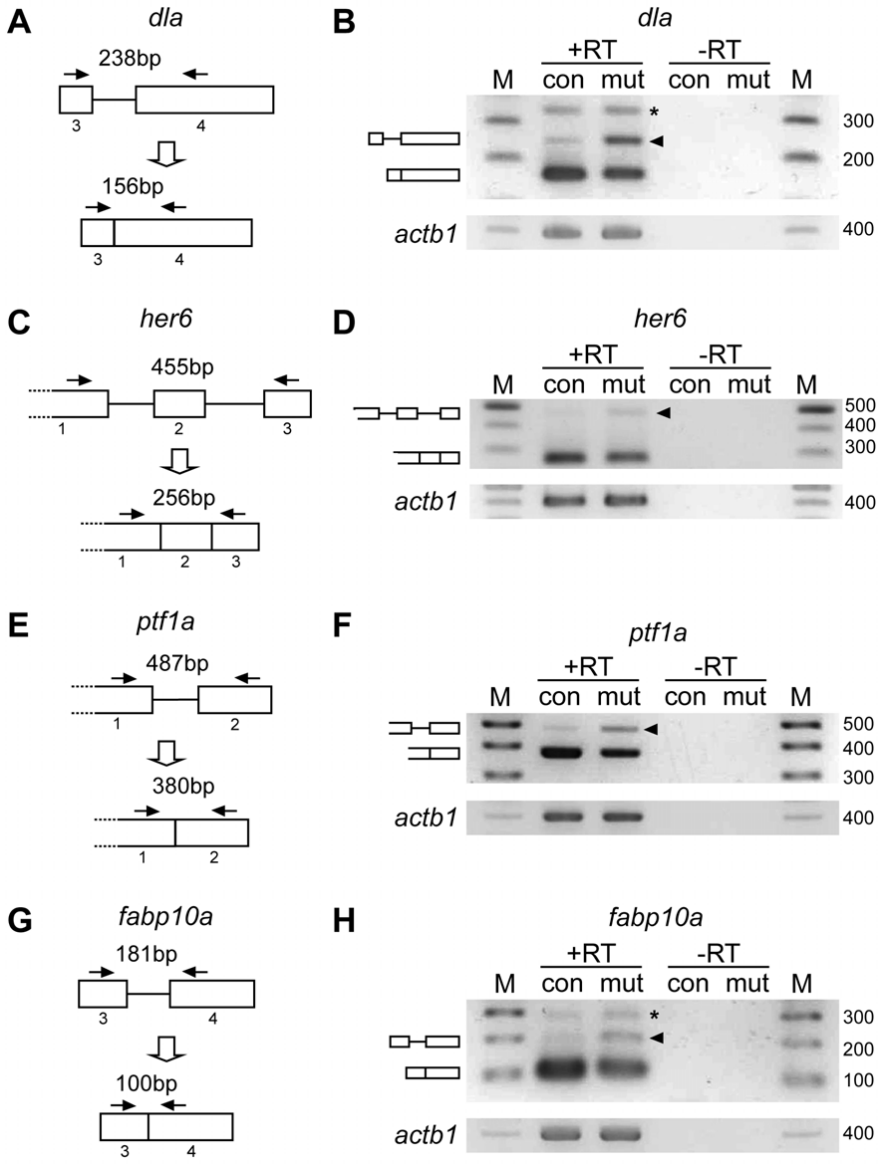
*Ddx46* deficiency affects pre-mRNA splicing in the digestive organs and brain. (A–H) Scheme of the *dla*, *her6*, *ptf1a*, and *fabp10a* pre-mRNA regions analyzed for splicing (boxes, exons; lines, introns; arrows, primers) (A, C, E, G). The splicing status of *dla*, *her6*, *ptf1a*, and *fabp10a* pre-mRNA was monitored using RT-PCR with the primers indicated in scheme A, C, E, and G, respectively. Unspliced *dla*, *her6*, *ptf1a*, and *fabp10a* mRNAs were retained in the *Ddx46^hi2137/hi2137^* mutant (mut) larvae compared to the control (con) larvae (arrowheads in B, D, F, H). Unspliced and spliced PCR products were verified by sequencing. +RT refers to the validation reaction itself, and −RT represents the respective control reaction without reverse transcriptase. *actb1* is a loading control by using primers designed in the exon 6. M, DNA size markers (sizes in bp); the asterisks point to nonspecific PCR products. Control larvae were sibling WT or *Ddx46^hi2137/+^* larvae and had normal phenotypes.

Because yeast Prp5 and human DDX46 are known to be involved in pre-mRNA splicing, we tested whether the *Ddx46* mutant had defects in this process. For the analyses of pre-mRNA splicing in the *Ddx46^hi2137/hi2137^* mutants, we examined the splicing status of four genes (*dla* and *her6* in the brain, and *fabp10a* and *ptf1a* in the digestive organs) by performing an RT-PCR analysis that is often used to detect unspliced forms of mRNAs [34]–[Bibr pone.0033675-Ros1] [36]. The analysis showed that the unspliced mRNAs were retained in the *Ddx46^hi2137/hi2137^* mutants at 3 or 4 dpf (Figure 6), suggesting that the pre-mRNA splicing process is defective in this mutant, as observed in yeast.

To test whether the effect on pre-mRNA splicing is restricted to a subset of genes or general, we further examined the pre-mRNA splicing of various genes, including housekeeping genes. Unspliced mRNAs of a housekeeping gene, *beta-2-microglobulin* (*b2m*) [37], were retained in the heads of *Ddx46^hi2137/hi2137^* mutants (Figure S9). In contrast, we found that the splicing of *actb1* in the heads of *Ddx46^hi2137/hi2137^* mutants was normal compared to that in the heads of control larvae (Figure S9). These results suggest that the effect of pre-mRNA splicing may be specific to a certain set of genes in the *Ddx46^hi2137/hi2137^* mutants.

## Discussion

### Functional significance of the ATPase domain and C-terminal region in Ddx46

All DExD/H-box proteins have nine conserved motifs, which are required for ATP binding and hydrolysis, RNA binding, and helicase activity [5]–[Bibr pone.0033675-Rocak1] [Bibr pone.0033675-Bleichert1] [8]. It has been clearly shown that the ATP hydrolysis of Prp5 is necessary for the stable association of U2 snRNP with pre-mRNA and pre-spliceosome formation in *S. cerevisiae* and *S. pombe* [6], [7]. In this study, our rescue experiments showed that the introduction of a point mutation into the ATPase domain of Ddx46, which disrupts the ATPase activity of *S. pombe* Prp5 (SpPrp5), leads to the loss of the rescue capability of Ddx46 for the *Ddx46^hi2137/hi2137^* mutant phenotype (Figure 3). Therefore, the ATP hydrolysis by the ATPase domain in Ddx46 may be required for the Ddx46 to function during zebrafish development.

In addition to the involvement of the ATPase domain, the role of the N-terminal region in Ddx46 has been reported in SpPrp5 and human DDX46 [10]. Both proteins physically associate with the U1 and U2 snRNPs through their N-terminal regions [10], when they function in pre-mRNA splicing. SpPrp5 contains distinct U1- and U2-interacting domains in its N-terminal region that are required for pre-spliceosome assembly [10]. In contrast to N-terminal region functioning, the function of the C-terminal region of Ddx46 proteins has not yet been analyzed. The alignment of the Ddx46 proteins of various vertebrates reveals high homology in the C-terminal region (see Figure S2), but to date, no specific motif has been reported in this region. Although the phenotype of the *mor^ha4^* mutant and our rescue experiments using a *mor^ha4^* mutant form of Ddx46 (Ddx46-I942S) indicated that the C-terminal region of Ddx46 is critical for its function in zebrafish development, further studies are needed to uncover the role of the Ddx46 C-terminal region and the influence of the *mor^ha4^* point mutation on Ddx46 function.

### *In vivo* function of zebrafish Ddx46 during development

In this study, we showed that the unspliced mRNAs of *dla*, *her6*, *ptf1a*, and *fabp10a* were retained in the *Ddx46^hi2137/hi2137^* mutant (Figure 6). We further showed that the splicing of the housekeeping gene *actb1*, but not *b2m*, was normal in the heads of *Ddx46^hi2137/hi2137^* mutants (Figure S9). These results, combined with functional analyses of yeast Prp5 and human DDX46, suggest that zebrafish Ddx46 may be required for pre-mRNA splicing during development, and that the effect of splicing may be specific to a certain set of genes in the affected organs. Since four genes (*dla*, *her6*, *fabp10a*, and *ptf1a*) were selected as simple markers for organ development, it is possible that the defects in the pre-mRNA splicing of genes other than these four lead to the phenotypes of the *Ddx46* mutant.

Assessment of pre-mRNA status by RT-PCR, which is not a quantitative analysis, showed that the spliced mRNAs of the five genes (*dla*, *her6*, *fabp10a*, *ptf1a*, and *b2m*) we tested were still present in the *Ddx46^hi2137/hi2137^* mutants (Figures 6 and S9). There are two possible explanations for this finding. One is that Ddx46 protein from the maternally inherited transcript may rescue pre-mRNA splicing. A previous study revealed that the maternally derived minichromosome maintenance protein 5 persists beyond 3 dpf in zebrafish larvae [38]. This indicates that a maternally derived protein is very stable during early development. Alternatively, it is possible that the five genes (*dla*, *her6*, *fabp10a*, *ptf1a*, and *b2m*) require Ddx46 for their splicing in a subset of tissues, but not in other tissues where splicing may occur in a Ddx46-independent manner. Detailed biochemical analyses will be needed to elucidate the *in vivo* function of Ddx46 during vertebrate development.

### Organ-specific requirement of Ddx46 in zebrafish development

Recent microarray profiling and expression cloning analyses have revealed that some housekeeping genes are expressed in specific tissues or organs, but others have shown ubiquitous expression during development [39], [40]. In zebrafish, microarray profiles and *in situ* analyses have shown that nucleolar genes, which are generally thought to be ubiquitously expressed, are preferentially expressed in the developing gastrointestinal tract [39]. Consistent with these results, zebrafish mutations in RNA polymerase III [41] and nucleolar protein RBM19 [42] showed specific defects in digestive organ formation during development because these genes are expressed in the digestive organs [41], [42].

Expression cloning screens in *Xenopus laevis* have revealed that some pre-mRNA splicing genes demostrate the tissue- or organ-specific expression and function analyzed using whole-mount *in situ* hybridization and overexpression experiments during development [40]. Moreover, analyses of zebrafish mutants and knockdown experiments have revealed the tissue-specific function of some splicing or splicing-related factors during vertebrate development: sublethal knockdown of the pre-mRNA processing factor 31 (Prpf31) predominantly affects retinal transcripts [43]; the *splicing factor proline/glutamine rich* (*sfpq*) gene, which is strongly expressed in the developing brain, is required for cell survival and neuronal development [44]; the *ubiquitin specific peptide 39* (*usp39*) gene is involved in embryonic pituitary homeostasis by regulating the *retinoblastoma 1* pre-mRNA splicing and *E2F transcription factor 4* expression [35]; and a mutation of a p110 protein, which functions in recycling of the U4/U6 snRNPs, leads to organ-specific defects during development [45].

These reports, combined with our results, suggest that some splicing genes may be specific to digestive organ and brain development in *X. laevis* and zebrafish. It is possible that another redundant DExD/H-box helicase functions in the pre-mRNA splicing in other tissues or organs, where *Ddx46* is not expressed. An alternative possibility is that some transcriptional/post-transcriptional genes, including splicing genes, are not specific to digestive organ and brain development. As observed with *Ddx46* expression during digestive organ and brain development, high expression levels of some transcriptional/post-transcriptional genes are needed to maintain a high number of cell cycles, because these organs grow particularly fast during larval stages. Further study will be necessary to elucidate the organ-specific requirement of Ddx46 in zebrafish development.

In summary, we demonstrated that a mutation in *Ddx46* is responsible for defects in the digestive organs and brain of the zebrafish mutant *mor^ha4^*. Consistent with the phenotype of *mor^ha4^* or *Ddx46* mutant larvae, the expression of *Ddx46* was gradually restricted to these organs as development proceeded after 2 dpf. Our rescue experiments revealed that both ATPase and the C-terminal domains of Ddx46 are necessary for its function. Based on our findings, we propose a model in which *Ddx46* is specifically expressed in the digestive organs and brain and is required for pre-mRNA splicing in these organs. Future investigations of the function of Ddx46 should lead to a better understanding of the splicing processes during vertebrate development.

## Materials and Methods

### Ethics statement

At present no approval needs to be given for research on zebrafish because in accordance with Ministry of Education, Culture, Sports, Science and Technology, Notice No. 71 (June 1, 2006) there is no rule on fish use at Hiroshima University.

### Zebrafish husbandry and N-ethyl-N-nitrosourea mutagenesis

Zebrafish were obtained from the Zebrafish International Resource Center (Oregon, USA). Adult zebrafish and zebrafish embryos were maintained under a 14-h day/ 10-h night cycle at 28.5°C. Embryos were incubated in 1/3 Ringer's solution (39 mM NaCl, 0.97 mM KCl, 1.8 mM CaCl_2_, 1.7 mM HEPES, pH 7.2) at 28.5°C and staged according to Kimmel et al. [46]. The *Ddx46* allele *hi2137* was isolated during an insertional mutagenesis screening [15] (http://web.mit.edu/hopkins/group11.html), and the *Ddx46^hi2137/+^* fish was obtained from the Zebrafish International Resource Center.

A *mor^ha4^* mutant was isolated during a mutagenesis screen performed in our laboratory. G0 males (AB strain) were mutagenized with N-ethyl-N-nitrosourea as described previously [47]. F1 progeny were grown from G0 males crossed to AB strain females. The F2 family was established by crossing F1 male and female fish. F3 larvae obtained by crossing pairs of F2 fish were fixed at 60 hpf and screened by whole-mount *in situ* hybridization for the expression of *foxa3* [18] (number of mutated genomes screened, 269). Families of larvae that showed abnormal expression of *foxa3* were subjected to further analyses.

### Positional cloning

The *mor* gene was mapped on a hybrid genetic background, AB/India, via bulked segregant analysis between microsatellite markers Z10508 and Z15212_1 on LG 21 [48]. Based on the Zv6 zebrafish genome database (http://www.ensembl.org/Danio_rerio/Info/Index), the closest marker, Z12027_1, was in the intron of the *Ddx46* gene The cDNA was prepared from pools of mutant or WT sibling larvae using RT-PCR with the following primers: 5′-GGAATTCGCGACAACATGGGCCGAGAG-3′ and 5′-CCCAAGCTTAGCAGAGAGCCAGAGGAGCG-3′, and was sequenced to find the mutation. To confirm that *Ddx46* was tightly linked to the *mor^ha4^* mutation, DNA fragments were amplified with the following PCR primers: 5′-TGTGTTGGCCTGAACGCTTG-3′ and 5′- AGACGTGACCTTCCACCTTG-3′. The amplified products were digested with *Mbo*I and resolved on 1% agarose gels. The *mor^ha4^* mutation abolished an *Mbo*I site.

### Whole-mount *in situ* hybridization, histology, genotyping, immunohistochemistry, and detection of cell death

Whole-mount *in situ* hybridizations and histological analyses were performed as described previously [49], [50], and riboprobes were prepared according to published instructions. For histological analysis, embryos were embedded in JB4 (Polysciences), and 7-µm sections were cut with a microtome and stained with hematoxylin and eosin. After whole-mount *in situ* hybridization and histological analyses, the *mor^ha4^* larvae were genotyped as described above. *Ddx46^hi2137^* mutants were confirmed with genotyping using two pairs of primers: one pair derived from the *LacZ* gene (5-ATCCTCTAGACTGCCATGG-3 and 5-ATCGTAACCGTGCATCTG-3), which is harbored by the viral vector, and the other derived from intron 1 of the *Ddx46* genomic sequence (5-GTGAGTTTACTGCTGCGACAAC-3 and 5-CTTGCGTTCTCTGGATCTGC-3), which flanks the viral vector insertion site.

Whole-mount immunohistochemistry for carboxypeptidase A was performed as described previously [32]. Rabbit anti-bovine carboxypeptidase A antibody (Rockland) and Alexa Fluor® 488 goat anti-rabbit IgG antibody (Invitrogen, Life Technologies Corp.) were used for the primary and secondary antibody, respectively. For detection of apoptotic cells, we performed TUNEL staining using an *in situ* Cell Death Detection Kit (Roche Diagnostics) according to the manufacturer's instructions. The stained embryos were embedded in 0.7% low-melting-temperature agarose gel in 1/3 Ringer's solution and imaged on an Olympus FV1000-D confocal microscope.

### mRNA injections

To introduce point mutations, we performed site-directed mutagenesis using a QuickChange Site-Directed Mutagenesis Kit (Stratagene), according to the manufacturer's instructions. The coding regions of *Ddx46-K402A* and *Ddx46-I942S* were verified by sequencing both strands. The pCS2+ vector carrying the cDNA fragment encoding *Ddx46*, *Ddx46-K402A*, *Ddx46-I942S*, or *egfp* was used in this study. Capped mRNA was synthesized using a SP6 mMESSAGE mMACHINE (Invitrogen, Life Technologies Corp.). For the overexpression experiments, *Ddx46*, *Ddx46-K402A*, *Ddx46-I942S*, or *egfp* mRNA (160 pg each) was injected at the one-cell stage.

### Northern blotting and RT-PCR analysis of splicing

Total RNA was prepared using TRIzol (Invitrogen, Life Technologies Corp.) from 40 or more pooled 3.5 dpf WT or *Ddx46^hi2137/hi2137^* mutant larvae that were identified morphologically or molecularly. Total RNA was separated using electrophoresis on a 1.0% agarose gel containing 4-morpholinopropanesulphonic acid and 2% formaldehyde, and blotted onto a nylon membrane (Amersham Hybond-N^+^, GE Healthcare). The RNA was fixed to the membrane via UV irradiation and probed with a DIG-labeled antisense RNA probe. Hybridization was performed in DIG Easy Hyb (Roche) at 65°C for 12 hours or more, and the signals were detected with CDP-Star (Roche), according to the manufacturer's instructions. The cDNA fragments for *Ddx46* and *actb1* were used as templates for the antisense probes.

For RT-PCR analyses of *dla*, *her6*, *fabp10a*, and *ptf1a*, total RNA was prepared from control and *Ddx46^hi2137/hi2137^* mutant larvae at 3 dpf or 4 dpf, as described above. For RT-PCR analyses of *actb1* (accession number, NM_131031) and *b2m* (transcript variant 1; accession number, NM_131163) [37], total RNA was prepared from the heads of 40 control and *Ddx46^hi2137/hi2137^* mutant larvae at 4 dpf. One microgram of DNase-treated RNA was reversetranscribed with oligo-d(T) (*dla*, *her6*, *fabp10a*, and *ptf1a*) or random 9mer (*actb1* and *b2m*) priming, and Reverse transcriptase XL (AMV) (TaKaRa). RT-PCR was performed to monitor splicing of *dla*, *her6*, *fabp10a*, *ptf1a*, *actb1*, and *b2m*. The primer pairs and detailed PCR conditions used to amplify each of these genes are listed in Tables S1 and S2.

## Supporting Information

Figure S1 **The size of the exocrine pancreas is reduced in the** ***mor^ha4^*** **mutant.** (A, B) High-power, lateral views of the immunostained exocrine pancreas from 5.5 dpf WT and *mor^ha4^* larvae. Both larvae were processed for carboxypeptidase A immunohistochemistry. The size of the exocrine pancreas was markedly reduced in the *mor^ha4^* mutant compared to the WT larva. Scale bars, 50 µm.
(TIF)

Figure S2 **The C-terminus region of Ddx46 is highly conserved among vertebrates.** Amino acid sequence alignment of the Ddx46 proteins of different vertebrates. The yellow, red, and orange boxes represent the N-terminal, DEAD-box helicase, and C-terminal domains, respectively. The C-terminal region of zebrafish Ddx46 was compared with those of human, mouse, and chicken Ddx46 proteins. Conserved amino acids in at least two species and similar amino acids are highlighted in black and gray, respectively. The red arrowhead and box indicate the mutated amino acid isoleucine found in the *mor^ha4^* mutant.
(TIF)

Figure S3 **Transheterozygote (** ***mor^ha4^*** **/** ***Ddx46^hi2137^*** **) of** ***mor^ha4^*** **and** ***Ddx46^hi2137^*** **shows the phenocopy of the** ***mor^ha4^*** **mutant.** (A–F) Lateral (A–D) and dorsal (E, F) views of live control and *mor^ha4^*/*Ddx46^hi2137^* larvae at 5 dpf. The swim bladder failed to inflate (arrows in A, B), the intestine lacked folds (arrowheads in C, D), and the retinae were reduced in size (brackets in E, F) in the *mor^ha4^*/*Ddx46^hi2137^* mutant. Conversely, somite formation in the *mor^ha4^*/*Ddx46^hi2137^* mutant appeared normal (arrowheads in A, B). Control larvae were sibling WT, *mor^ha4/+^* or *Ddx46^hi2137/+^* larvae and had normal phenotypes.
(TIF)

Figure S4 **Expression of** ***foxa3*** **is unaffected in the** ***Ddx46^hi2137/hi2137^*** **mutant at 2.5 dpf.** (A, B) Expression of *foxa3* was examined using whole-mount *in situ* hybridization. Dorsal views, anterior to the top. The *foxa3* expression in control larvae (A) was indistinguishable from that in the *Ddx46^hi2137/hi2137^* mutant (B) at 2.5 dpf. Control larvae were sibling WT or *Ddx46^hi2137/+^* larvae and had normal phenotypes.
(TIF)

Figure S5 **Expression of** ***myod1*** **is normal in the** ***Ddx46^hi2137/hi2137^*** **mutant.** (A, B) Expression of *myod1* was examined using whole-mount *in situ* hybridization. Lateral views, anterior to the left. The *myod1* expression in control larvae (A) was indistinguishable from that in the *Ddx46^hi2137/hi2137^* mutant (B) at 3.5 dpf. Control larvae were sibling WT or *Ddx46^hi2137/+^* larvae and had normal phenotypes.
(TIF)

Figure S6 **Expression of molecular markers for digestive organs and brain is reduced in the** ***mor^ha4/ha4^*** **mutant.** (A–B) The expression of *dla* was examined using whole-mount *in situ* hybridization at 3 dpf. All lateral views, anterior to the left. (C–J) The expression of *fabp2*, *fabp10a*, *ptf1a*, and *ins* was examined using whole-mount *in situ* hybridization at 3.5 dpf. All dorsal views, anterior to the top. Although the expression of *dla*, *fabp2*, and *fabp10a* was slightly reduced, the *ptf1a* expression was markdly reduced at 3 or 3.5 dpf in the *mor^ha4/ha4^* mutants (A–H). In contrast, the *ins* expression in the *mor^ha4/ha4^* mutant did not change at these developmental stages (I, J). Control larvae were sibling WT or *mor^ha4/+^* larvae and had normal phenotypes.
(TIF)

Figure S7 **Expression of molecular markers for digestive organs and brain is also reduced in the transheterozygote** ***mor^ha4^*** **/** ***Ddx46^hi2137^*** **mutant.** (A, B) The expression of *dla* was examined using whole-mount *in situ* hybridization at 3 dpf. All lateral views, anterior to the left. (C–H) The expression of *fabp10a*, *ptf1a*, and *ins* was examined by whole-mount *in situ* hybridization at 3.5 dpf. All dorsal views, anterior to the top. The intensity and area of *dla*, *fabp10a*, and *ptf1a* expression were markedly reduced at 3 or 3.5 dpf in the *mor^ha4^*/*Ddx46^hi2137^* mutants. In contrast, *ins* expression in this transheterozygote was unchanged at these developmental stages. These phenotypes are the same as those of the *Ddx46^hi2137/hi2137^* mutant. Control larvae were sibling WT, *mor^ha4/+^*, or *Ddx46^hi2137/+^* larvae and had normal phenotypes.
(TIF)

Figure S8 **Expression of various molecular markers for digestive organs and brain is reduced in the** ***Ddx46^hi2137/hi2137^*** **mutant.** (A–F) The expression of *her4*, *neurog1*, and *neurod* for brain was examined using whole-mount *in situ* hybridization at 3 dpf. All lateral views, anterior to the left. (G–N) The expression of *hlxb9la*, *cpa5*, *gata6*, and *dhrs9* for digestive organs was examined using whole-mount *in situ* hybridization at 3.5 dpf. All dorsal views, anterior to the top. In the *Ddx46^hi2137/hi2137^* mutants, the intensity and area of all of these gene expressions were markedly reduced at 3 or 3.5 dpf. Control larvae were sibling WT or *Ddx46^hi2137/+^* larvae and had normal phenotypes.
(TIF)

Figure S9 **Pre-mRNA splicing of the housekeeping gene** ***actb1*** **, but not** ***b2m*** **, is unaffected in the** ***Ddx46^hi2137/hi2137^*** **mutant.** (A–D) Scheme of the *b2m* and *actb1* pre-mRNA regions analyzed for splicing (boxes, exons; lines, introns; arrows, primers) (A, C). The splicing status of *b2m* and *actb1* pre-mRNA was monitored using RT-PCR with the primers indicated in scheme A and C, respectively. Total RNA was isolated from the heads of *Ddx46^hi2137/hi2137^* mutants (mut) and control (con) larvae. Unspliced *b2m* mRNAs were retained in the *Ddx46^hi2137/hi2137^* mutants compared to the control larvae (arrowheads in B), whereas the splicing of *actb1* was unaffected in the *Ddx46^hi2137/hi2137^* mutants (arrowheads in D). Unspliced and spliced PCR products were verified by sequencing. +RT refers to the validation reaction itself, and −RT represents the respective control reaction without reverse transcriptase. 18S rRNA was used as a loading control. M, DNA size markers (sizes in bp). Control larvae were sibling WT or *Ddx46^hi2137/+^* larvae and had normal phenotypes.
(TIF)

Table S1 The list and sequence of primers used for RT-PCR analysis.
(XLS)

Table S2 PCR thermal cycler program for RT-PCR.
(XLS)
