# Supplementary figures and images for: DEAD-Box Protein Ddx46 Is Required for the Development of the Digestive Organs and Brain in Zebrafish
Source: PLoS One. 2012 Mar 19;7(3):e33675. doi: 10.1371/journal.pone.0033675 (PMC3307747; doi:10.1371/journal.pone.0033675)

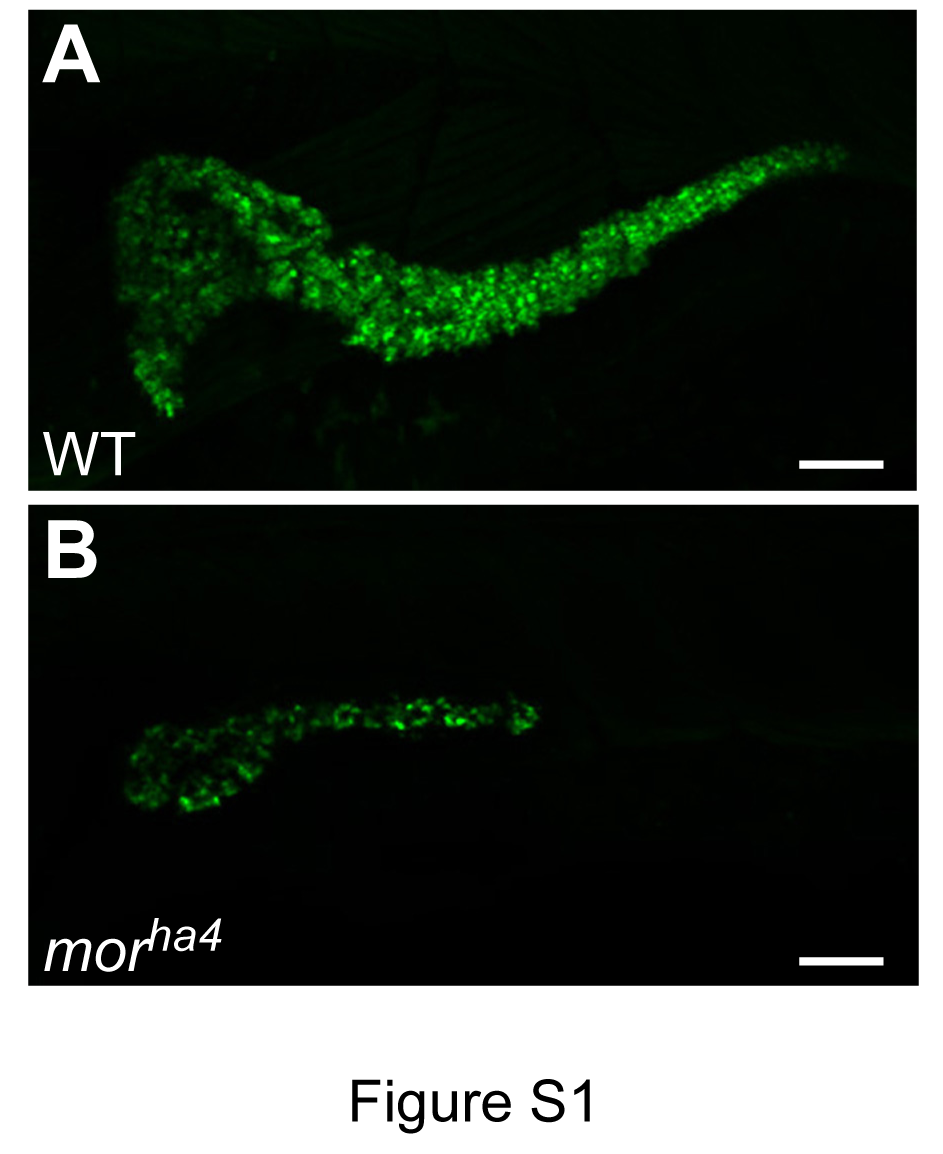

Supplement: Figure S1 — The size of the exocrine pancreas is reduced in the morha4 mutant. (A, B) High-power, lateral views of the immunostained exocrine pancreas from 5.5 dpf WT and morha4 larvae. Both larvae were processed for carboxypeptidase A immunohistochemistry. The size of the exocrine pancreas was markedly reduced in the morha4 mutant compared to the WT larva. Scale bars, 50 µm. (TIF) [file pone.0033675.s001.tif]

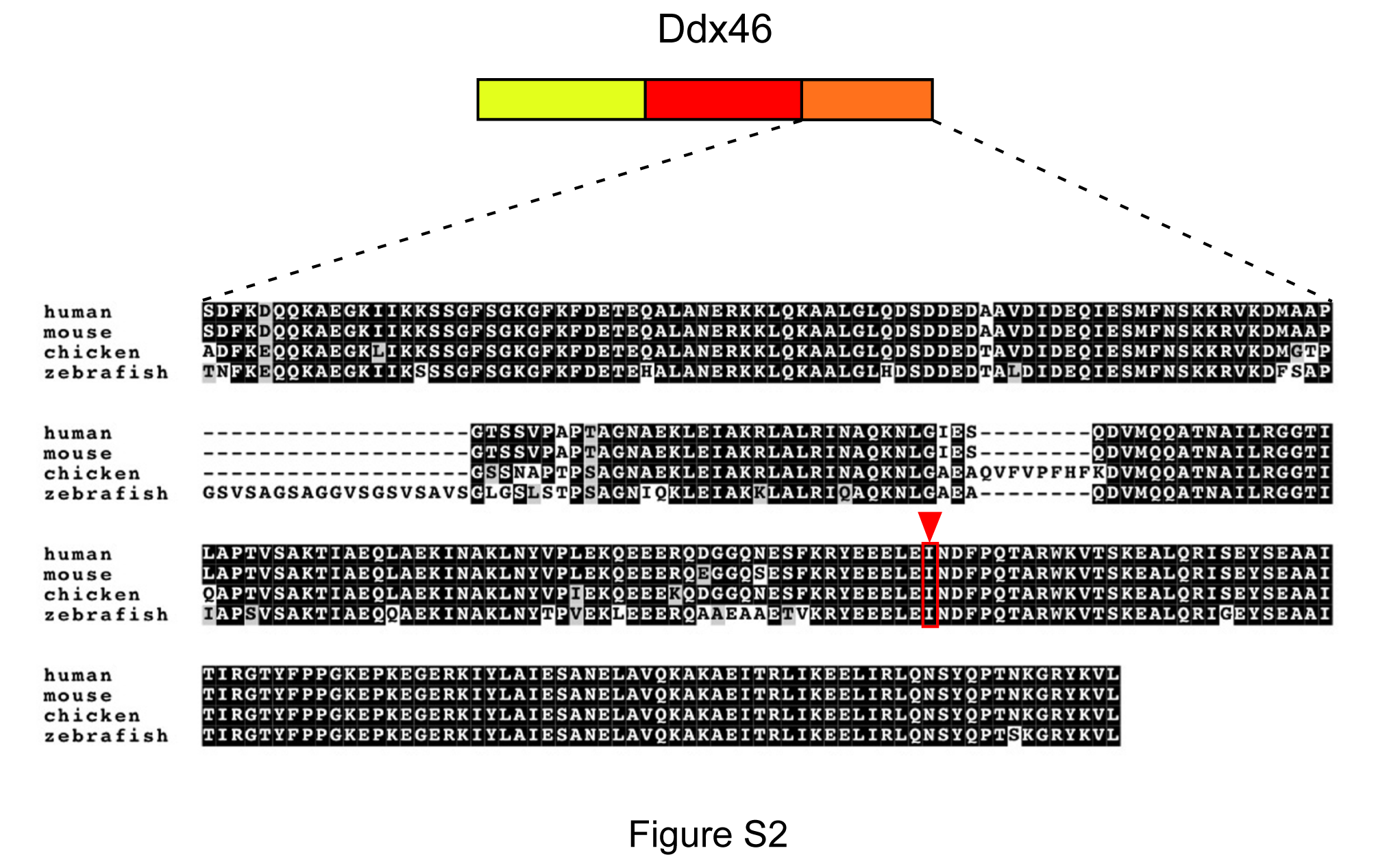

Supplement: Figure S2 — The C-terminus region of Ddx46 is highly conserved among vertebrates. Amino acid sequence alignment of the Ddx46 proteins of different vertebrates. The yellow, red, and orange boxes represent the N-terminal, DEAD-box helicase, and C-terminal domains, respectively. The C-terminal region of zebrafish Ddx46 was compared with those of human, mouse, and chicken Ddx46 proteins. Conserved amino acids in at least two species and similar amino acids are highlighted in black and gray, respectively. The red arrowhead and box indicate the mutated amino acid isoleucine found in the morha4 mutant. (TIF) [file pone.0033675.s002.tif]

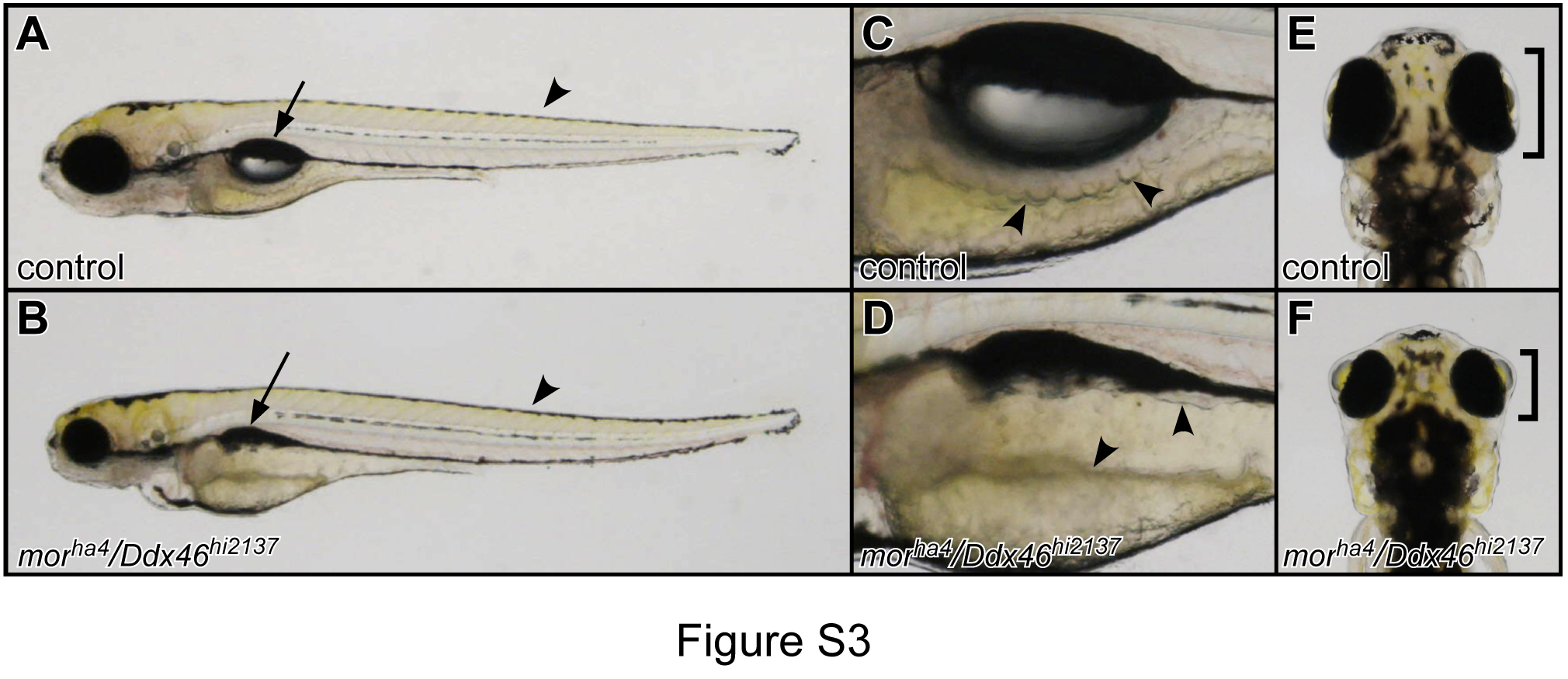

Supplement: Figure S3 — Transheterozygote ( morha4 / Ddx46hi2137 ) of morha4 and Ddx46hi2137 shows the phenocopy of the morha4 mutant. (A–F) Lateral (A–D) and dorsal (E, F) views of live control and morha4/Ddx46hi2137 larvae at 5 dpf. The swim bladder failed to inflate (arrows in A, B), the intestine lacked folds (arrowheads in C, D), and the retinae were reduced in size (brackets in E, F) in the morha4/Ddx46hi2137 mutant. Conversely, somite formation in the morha4/Ddx46hi2137 mutant appeared normal (arrowheads in A, B). Control larvae were sibling WT, morha4/+ or Ddx46hi2137/+ larvae and had normal phenotypes. (TIF) [file pone.0033675.s003.tif]

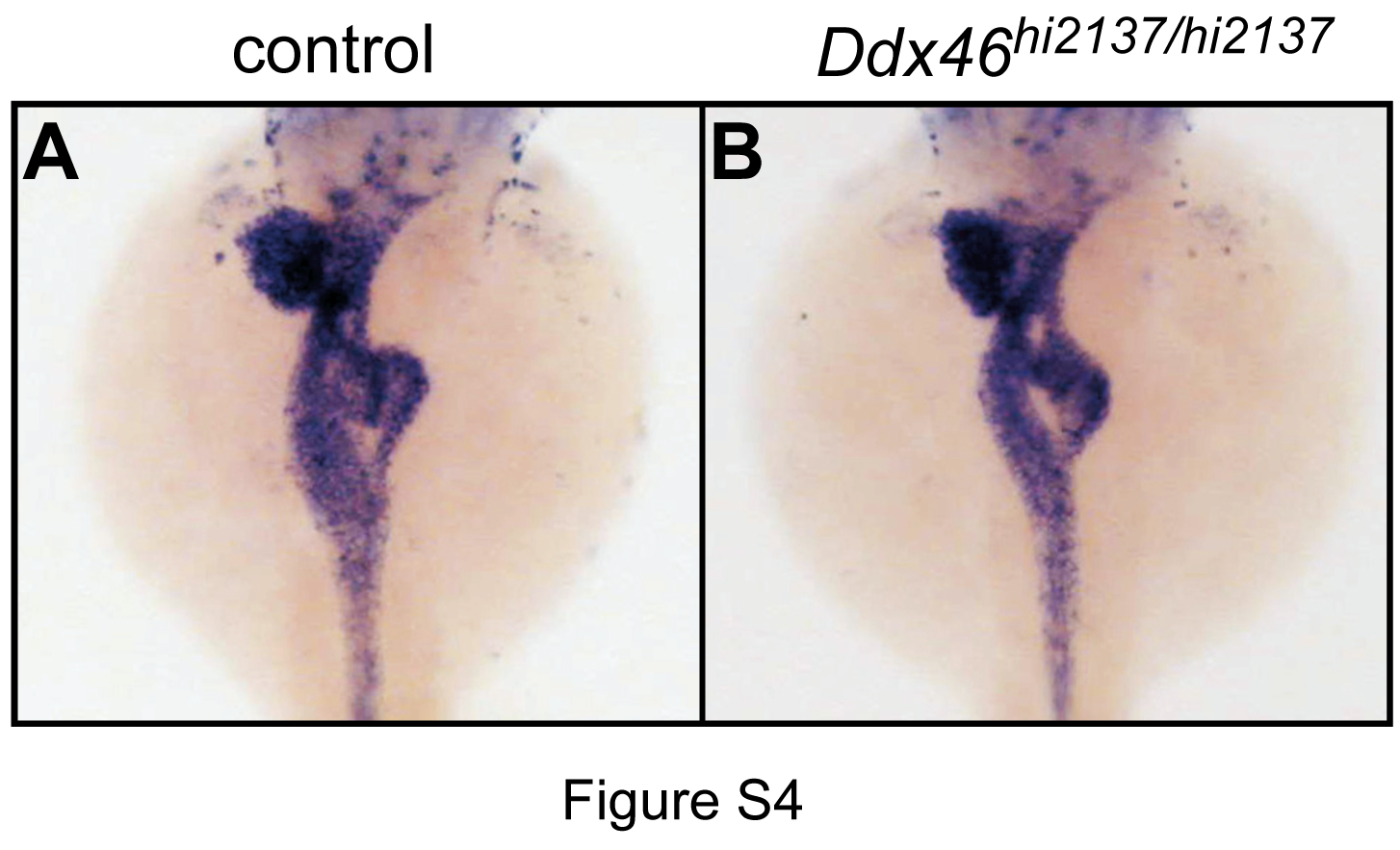

Supplement: Figure S4 — Expression of foxa3 is unaffected in the Ddx46hi2137/hi2137 mutant at 2.5 dpf. (A, B) Expression of foxa3 was examined using whole-mount in situ hybridization. Dorsal views, anterior to the top. The foxa3 expression in control larvae (A) was indistinguishable from that in the Ddx46hi2137/hi2137 mutant (B) at 2.5 dpf. Control larvae were sibling WT or Ddx46hi2137/+ larvae and had normal phenotypes. (TIF) [file pone.0033675.s004.tif]

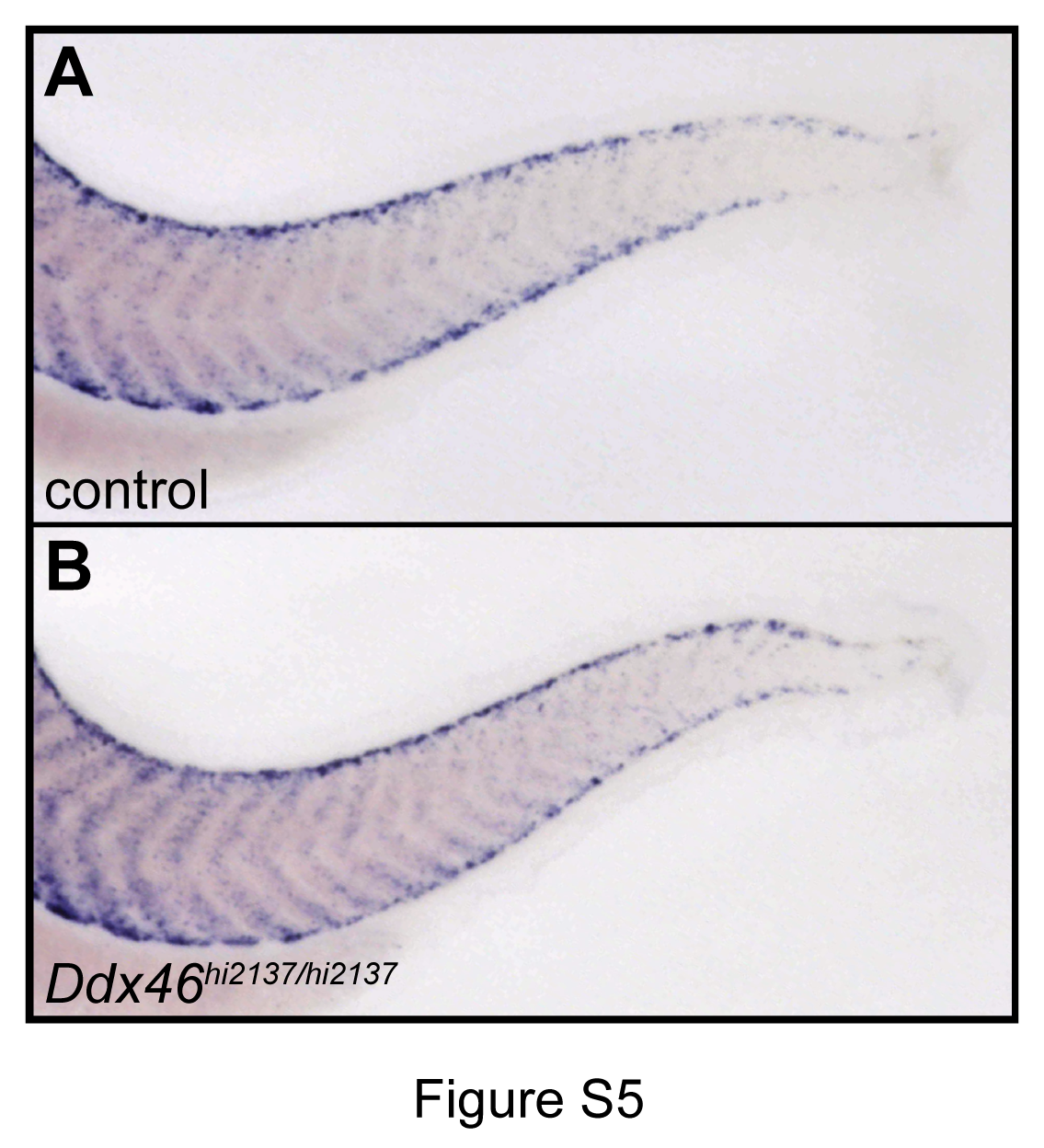

Supplement: Figure S5 — Expression of myod1 is normal in the Ddx46hi2137/hi2137 mutant. (A, B) Expression of myod1 was examined using whole-mount in situ hybridization. Lateral views, anterior to the left. The myod1 expression in control larvae (A) was indistinguishable from that in the Ddx46hi2137/hi2137 mutant (B) at 3.5 dpf. Control larvae were sibling WT or Ddx46hi2137/+ larvae and had normal phenotypes. (TIF) [file pone.0033675.s005.tif]

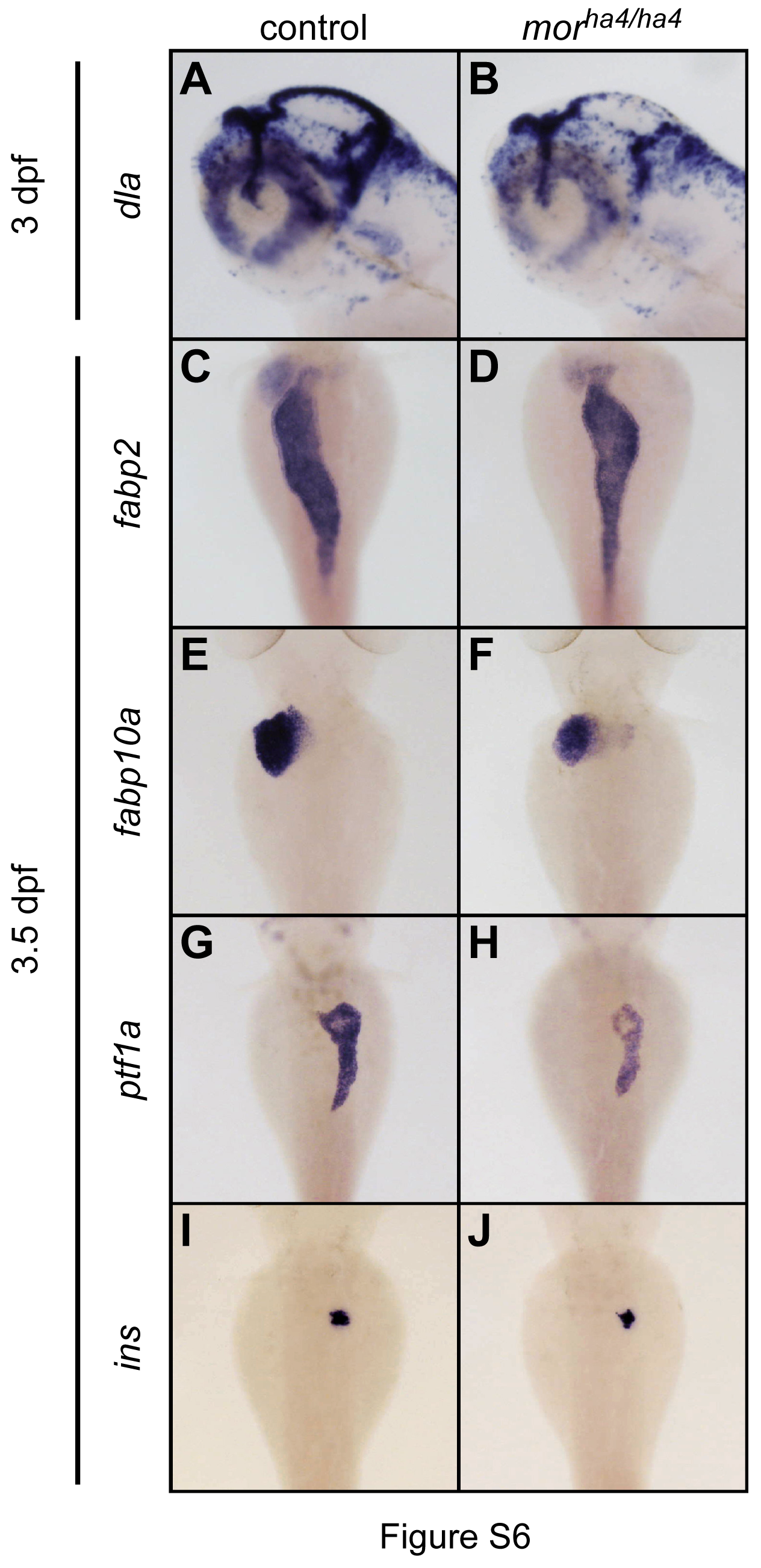

Supplement: Figure S6 — Expression of molecular markers for digestive organs and brain is reduced in the morha4/ha4 mutant. (A–B) The expression of dla was examined using whole-mount in situ hybridization at 3 dpf. All lateral views, anterior to the left. (C–J) The expression of fabp2, fabp10a, ptf1a, and ins was examined using whole-mount in situ hybridization at 3.5 dpf. All dorsal views, anterior to the top. Although the expression of dla, fabp2, and fabp10a was slightly reduced, the ptf1a expression was markdly reduced at 3 or 3.5 dpf in the morha4/ha4 mutants (A–H). In contrast, the ins expression in the morha4/ha4 mutant did not change at these developmental stages (I, J). Control larvae were sibling WT or morha4/+ larvae and had normal phenotypes. (TIF) [file pone.0033675.s006.tif]

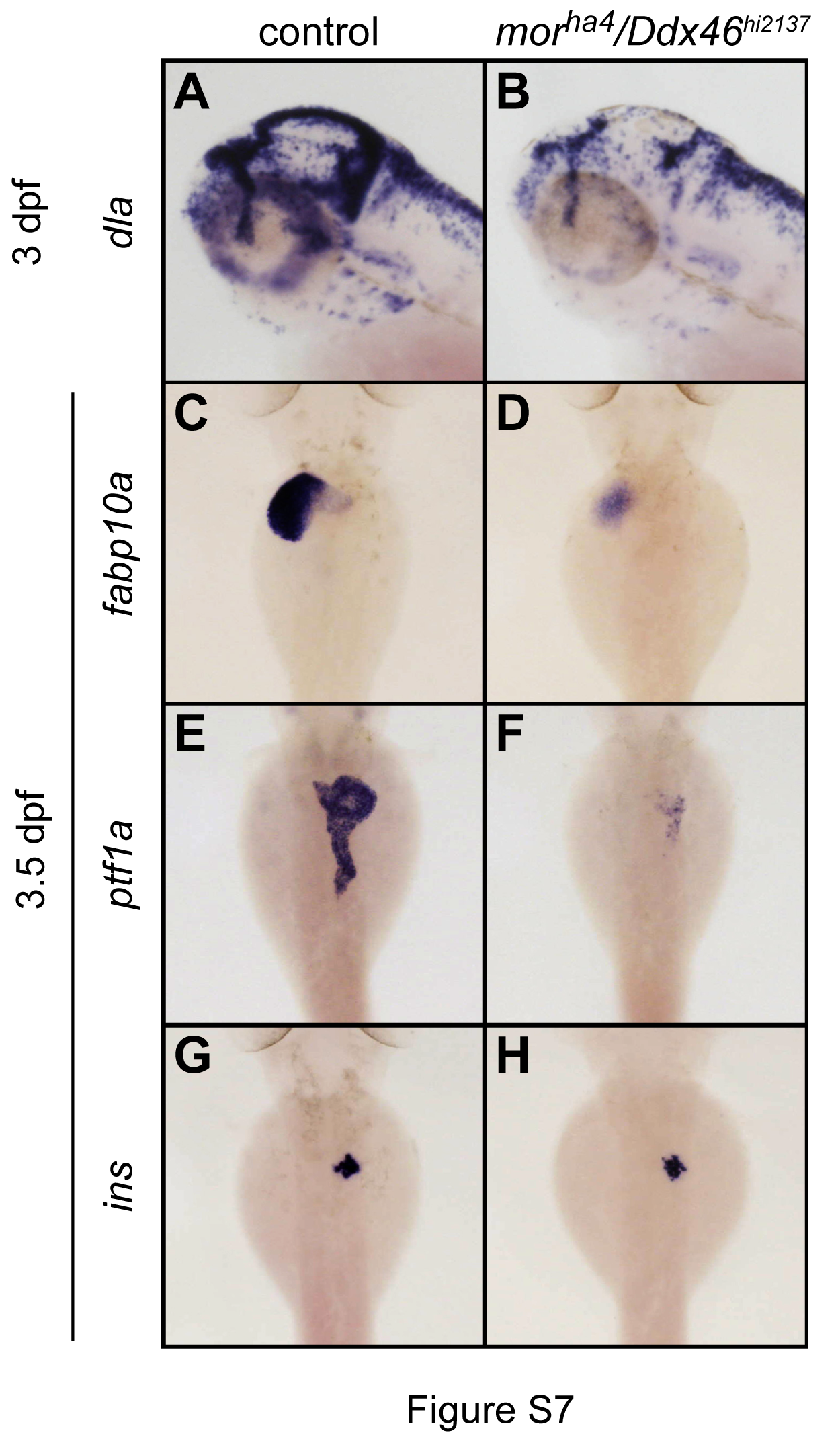

Supplement: Figure S7 — Expression of molecular markers for digestive organs and brain is also reduced in the transheterozygote morha4 / Ddx46hi2137 mutant. (A, B) The expression of dla was examined using whole-mount in situ hybridization at 3 dpf. All lateral views, anterior to the left. (C–H) The expression of fabp10a, ptf1a, and ins was examined by whole-mount in situ hybridization at 3.5 dpf. All dorsal views, anterior to the top. The intensity and area of dla, fabp10a, and ptf1a expression were markedly reduced at 3 or 3.5 dpf in the morha4/Ddx46hi2137 mutants. In contrast, ins expression in this transheterozygote was unchanged at these developmental stages. These phenotypes are the same as those of the Ddx46hi2137/hi2137 mutant. Control larvae were sibling WT, morha4/+, or Ddx46hi2137/+ larvae and had normal phenotypes. (TIF) [file pone.0033675.s007.tif]

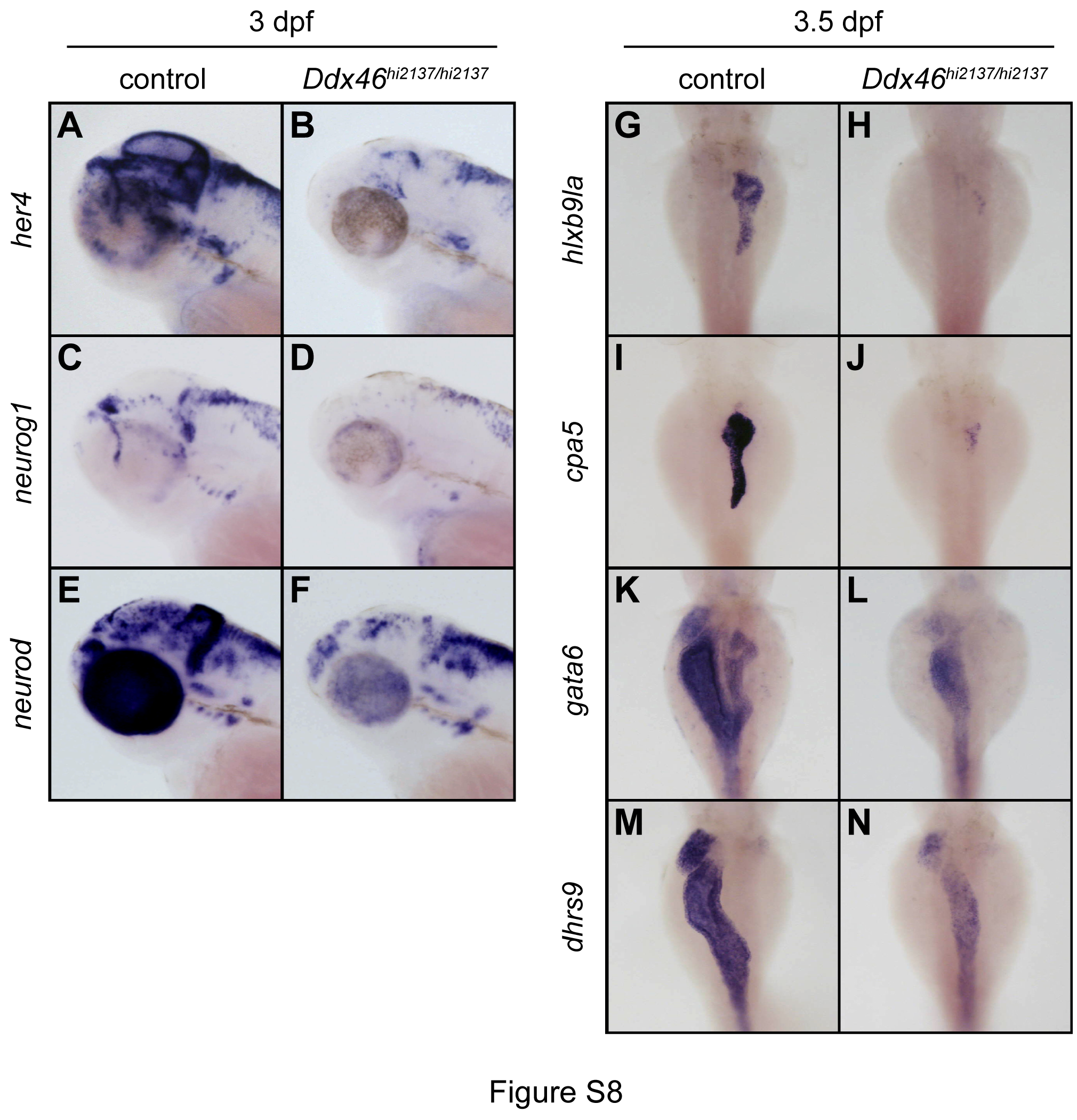

Supplement: Figure S8 — Expression of various molecular markers for digestive organs and brain is reduced in the Ddx46hi2137/hi2137 mutant. (A–F) The expression of her4, neurog1, and neurod for brain was examined using whole-mount in situ hybridization at 3 dpf. All lateral views, anterior to the left. (G–N) The expression of hlxb9la, cpa5, gata6, and dhrs9 for digestive organs was examined using whole-mount in situ hybridization at 3.5 dpf. All dorsal views, anterior to the top. In the Ddx46hi2137/hi2137 mutants, the intensity and area of all of these gene expressions were markedly reduced at 3 or 3.5 dpf. Control larvae were sibling WT or Ddx46hi2137/+ larvae and had normal phenotypes. (TIF) [file pone.0033675.s008.tif]

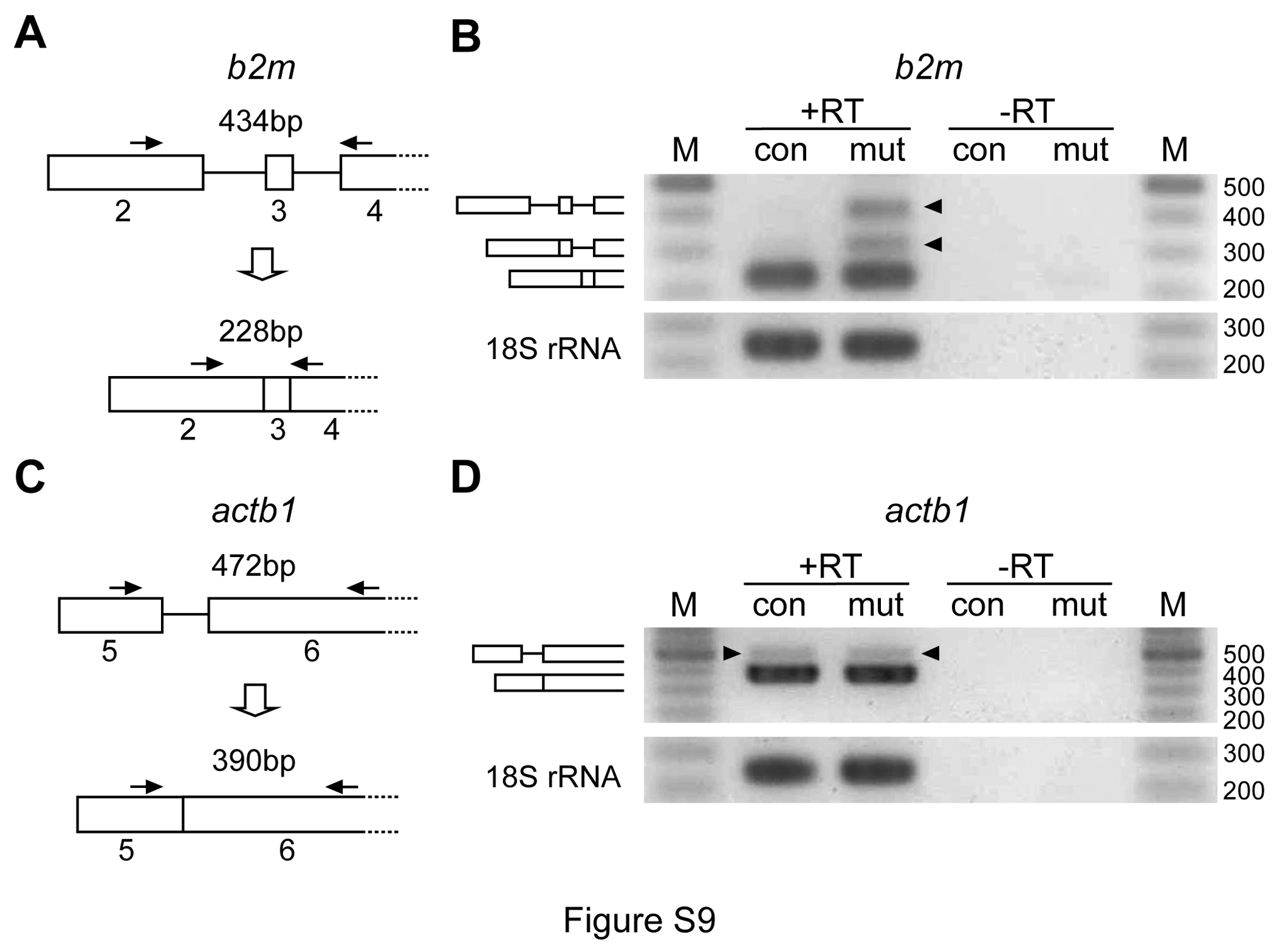

Supplement: Figure S9 — Pre-mRNA splicing of the housekeeping gene actb1 , but not b2m , is unaffected in the Ddx46hi2137/hi2137 mutant. (A–D) Scheme of the b2m and actb1 pre-mRNA regions analyzed for splicing (boxes, exons; lines, introns; arrows, primers) (A, C). The splicing status of b2m and actb1 pre-mRNA was monitored using RT-PCR with the primers indicated in scheme A and C, respectively. Total RNA was isolated from the heads of Ddx46hi2137/hi2137 mutants (mut) and control (con) larvae. Unspliced b2m mRNAs were retained in the Ddx46hi2137/hi2137 mutants compared to the control larvae (arrowheads in B), whereas the splicing of actb1 was unaffected in the Ddx46hi2137/hi2137 mutants (arrowheads in D). Unspliced and spliced PCR products were verified by sequencing. +RT refers to the validation reaction itself, and −RT represents the respective control reaction without reverse transcriptase. 18S rRNA was used as a loading control. M, DNA size markers (sizes in bp). Control larvae were sibling WT or Ddx46hi2137/+ larvae and had normal phenotypes. (TIF) [file pone.0033675.s009.tif]
